# Kernel Recursive Least-Squares Temporal Difference Algorithms with Sparsification and Regularization

**DOI:** 10.1155/2016/2305854

**Published:** 2016-06-29

**Authors:** Chunyuan Zhang, Qingxin Zhu, Xinzheng Niu

**Affiliations:** ^1^School of Computer Science and Engineering, University of Electronic Science and Technology of China, Chengdu 611731, China; ^2^College of Information Science and Technology, Hainan University, Haikou 570228, China

## Abstract

By combining with sparse kernel methods, least-squares temporal difference (LSTD) algorithms can construct the feature dictionary automatically and obtain a better generalization ability. However, the previous kernel-based LSTD algorithms do not consider regularization and their sparsification processes are batch or offline, which hinder their widespread applications in online learning problems. In this paper, we combine the following five techniques and propose two novel kernel recursive LSTD algorithms: (i) online sparsification, which can cope with unknown state regions and be used for online learning, (ii) *L*_2_ and *L*_1_ regularization, which can avoid overfitting and eliminate the influence of noise, (iii) recursive least squares, which can eliminate matrix-inversion operations and reduce computational complexity, (iv) a sliding-window approach, which can avoid caching all history samples and reduce the computational cost, and (v) the fixed-point subiteration and online pruning, which can make *L*_1_ regularization easy to implement. Finally, simulation results on two 50-state chain problems demonstrate the effectiveness of our algorithms.

## 1. Introduction

The least-squares temporal difference (LSTD) learning may be the most popular approach for policy evaluation in reinforcement learning (RL) [1, 2]. Compared with the standard temporal difference (TD) learning, LSTD uses samples more efficiently and eliminates all step-size parameters. However, LSTD also has some drawbacks. First, LSTD requires a matrix-inversion operation at each time step. To reduce computational complexity, Bradtke and Barto proposed a recursive LSTD (RLSTD) algorithm [1], and Xu et al. proposed a RLSTD(*λ*) algorithm [3]. But these two algorithms still require many features especially for highly nonlinear RL problems, since the RLS approximator assumes a linear model [4]. Second, when the number of features is larger than the number of training samples, LSTD is prone to overfitting. To overcome this problem, Kolter and Ng proposed an *L*_1_-regularized LSTD algorithm called LARS-TD for feature selection [5], but it is only applicable for batch learning and its implementation is complicated. On this basis, Chen et al. proposed an *L*_2_-regularized RLSTD algorithm [6]. In contrast with LARS-TD, it has an analytical solution, but it cannot obtain a sparse solution. Third, LSTD requires users to design the feature vector manually, and poor design choices can result in estimates that diverge from the optimal value function [7].

In the last two decades, kernel methods have been intensively and extensively studied in supervised and unsupervised learning [8]. The basic idea behind kernel methods can be summarized as follows: By using a nonlinear transform, the origin input data can be mapped into a high-dimensional feature space, and an inner product in this space can be interpreted as a Mercer kernel function. Thus, as long as a linear algorithm can be formulated in terms of inner products, there is no need to perform computations in the high-dimensional feature space [9]. Recently, there have also been many research works on kernelizing least-squares algorithms [9–13]. Here, we only review some works related to our proposed algorithms. One typical work is the sparse kernel recursive least-squares (SKRLS) algorithm with the approximate linear dependency (ALD) criterion [11]. Compared with traditional RLS algorithms, it not only has a good nonlinear approximation ability but also can construct the feature dictionary automatically. Similarly, Chen et al. proposed an *L*_2_-regularized SKRLS algorithm with the online vector quantization [12]. Besides having the good properties of SKRLS-ALD, it can avoid overfitting. In addition, Chen et al. proposed an *L*_1_-regularized SKRLS algorithm with the fixed-point subiteration [13], which can yield a much sparser dictionary.

Intuitively, we can also bring the benefits of kernel machine learning to LSTD algorithms. In fact, kernel-based RL algorithms have become more and more popular in recent years [14–22], and several works have been done for kernelizing LSTD algorithms. In an earlier paper, Xu proposed a sparse kernel-based LSTD(*λ*) (SKLSTD(*λ*)) algorithm with the ALD criterion [19]. Although this algorithm can avoid selecting features manually, it is only applicable for batch learning and its derivation is complicated. After that, Xu et al. proposed an incremental version of the SKLSTD(*λ*) algorithm for policy iteration [20], but this algorithm still requires a matrix-inversion operation at each time step. Moreover, the feature dictionary is required to be constructed offline, which makes this algorithm only approximate the value function correctly in the area of the state space that is covered by the training samples. Recently, Jakab and Csató proposed a sparse kernel RLSTD (SKRLSTD) algorithm by using a proximity graph sparsification method [21]. Unfortunately, its sparsification process is also offline. In addition, all of these algorithms do not consider regularization, whereas many real problems exhibit noise and the high expressiveness of the kernel matrix can result in overfitting [22].

In this paper, we propose two online SKRLSTD algorithms with *L*_2_ and *L*_1_ regularization, called OSKRLSTD-*L*_2_ and OSKRLSTD-*L*_1_, respectively. Compared with the derivation of SKLSTD(*λ*), our derivation uses Bellman operator along with projection operator and thus is more simple. To cope with unknown state-space regions and avoid overfitting, our algorithms use online sparsification and regularization techniques. Besides, to reduce computational complexity and avoid caching all history samples, our algorithms also use the recursive least-squares and the sliding-window technique. Moreover, different from LARS-TD, OSKRLSTD-*L*_1_ uses the subiteration and online pruning to find the fixed point. These techniques make our algorithms more suitable for online RL problems with a large or continuous state space. The rest of this paper is organized as follows. In Section 2, we present preliminaries and review the LSTD algorithm.  Section 3 contains the main contribution of this paper: we derive OSKRLSTD-*L*_2_ and OSKRLSTD-*L*_1_ algorithms in detail. In Section 4, we demonstrate the effectiveness of our algorithms for two 50-state chain problems. Finally, we conclude the paper in Section 5.

## 2. Background

In this section, we introduce the basic definitions and notations, which will be used throughout the paper without any further mention. We also review the LSTD algorithm, which is needed to establish our algorithms described in Section 3.

### 2.1. Preliminaries

In RL and dynamic programming (DP), an underlying sequential decision-making problem is often modeled as a Markov decision process (MDP). An MDP can be defined as a tuple *ℳ* = 〈*𝒮*, *𝒜*, *P*, *r*, *γ*, *d*〉 [5], where *𝒮* is a set of states, *𝒜* is a set of actions, *P* : *𝒮* × *𝒜* × *𝒮* → [0,1] is a state transition probability function where *P*(**s**, **a**, **s**′) denotes the probability of transitioning to state **s**′ when taking action **a** in state **s**, *r* ∈ *ℝ* is a reward function, *γ* ∈ [0,1] is the discount factor, and *d* is an initial state distribution. For simplicity of presentation, we assume that *𝒮* and *𝒜* are finite. Given an MDP *ℳ* and a policy *π* : *𝒮* → *𝒜*, the sequence **s**_1_, *r*_1_, **s**_2_, *r*_2_,… is a Markov reward process *ℛ* = 〈*𝒮*, *P* ^*π*^, *R* ^*π*^, *γ*, *d*〉, where *P* ^*π*^(**s**, **s**′) = ∑_**a**∈*𝒜*_ *π*(**a**∣**s**)*P*(**s**, **a**, **s**′) and *R* ^*π*^(**s**) = ∑_**a**∈*𝒜*_ *π*(**a**∣**s**)∑_**s**′∈*𝒮*_ *P*(**s**, **a**, **s**′)*r*(**s**, **a**, **s**′).

RL and DP often use the state-value function *V* ^*π*^(**s**) to evaluate how good the policy *π* is for the agent to be in state **s**. For an MDP, *V* ^*π*^(**s**) can be defined as *V* ^*π*^(**s**) = **E**_*π*_[∑_*t*=0_^*∞*^ *γ* ^*t*^ *r*_*t*_∣**s**_0_ = **s**], which must obey the Bellman equation [23],

(1) $$\begin{array}{c} V^{\pi }\left(\;s\;\right)=R^{\pi }\left(\;s\;\right)+\gamma \underset{s^{\prime }\in S}{\sum }P^{\pi }\left(\;s,s^{\prime }\;\right)V^{\pi }\left(\;s^{\prime }\;\right), \end{array}$$

or be expressed in vector form,

(2) $$\begin{array}{c} V^{\pi }=R^{\pi }+\gamma P^{\pi }V^{\pi }. \end{array}$$

If *P* ^*π*^ and *R* ^*π*^ are known, *V* ^*π*^ can be solved analytically; that is,

(3) $$\begin{array}{c} V^{\pi }={\left(\;I-\gamma P^{\pi }\;\right)}^{-1}R^{\pi }, \end{array}$$

where **I** is the |*𝒮* | ×|*𝒮*| identity matrix.

However, different from the case in DP, *P* ^*π*^ and *R* ^*π*^ are unknown in RL. The agent has to estimate *V* ^*π*^ by exploring the environment. Furthermore, many real problems have a large or continuous state space, which makes *V* ^*π*^(**s**) hard to be expressed explicitly. To overcome this problem, we often resort to linear function approximation; that is,

(4) V^πs=wTϕsor  V^π=Φw,

where **w** ∈ *ℝ* ^*m*^ is a parameter vector, **ϕ**(**s**) ∈ *ℝ* ^*m*^ is the feature vector of state **s**, and Φ = [**ϕ**(**s**_1_),…, **ϕ**(**s**_|*𝒮*|_)]^*T*^ is a |*𝒮* | × *m* feature matrix. Unfortunately, when approximating *V* ^*π*^ in this manner, there is usually no way to satisfy the Bellman equation exactly, because *R* ^*π*^ + *γP* ^*π*^Φ**w** may lie outside the span of Φ [5].

### 2.2. LSTD Algorithm

The LSTD algorithm presents an efficient way to find **w** such that ${\hat{V}}^{\pi }$ “approximately” satisfies the Bellman equation [5]. By solving the least-squares problem min_**u**∈*ℝ*^*m*^_‖Φ**u** − (*R* ^*π*^ + *γP* ^*π*^Φ**w**)‖_**D**_^2^, we can find a closest approximation Φ**u** ^*∗*^ in the span of Φ to replace *R* ^*π*^ + *γP* ^*π*^Φ**w**. Then, from (2) and (4), we can use **w** = **u** ^*∗*^ for approximating *V* ^*π*^. That means we can find **w** by solving the fixed-point equation:

(5) $$\begin{array}{c} w=f\left(\;w\;\right)=\underset{u\in R^{m}}{\arg\min}{\left\|\;\Phi u-\left(\;R^{\pi }+\gamma P^{\pi }\Phi w\;\right)\;\right\|}_{D}^{2}, \end{array}$$

where **D** is a nonnegative diagonal matrix indicating a distribution over states. Nevertheless, since *P* ^*π*^ and *R* ^*π*^ are unknown and since Φ is too large to form anyway in a large or continuous state space, we cannot solve (5) exactly. Instead, given a trajectory *T*_*t*_^*π*^ = {(**s**_*i*_, **s**_*i*_′, *r*_*i*_)∣*i* = 1,…, *t*} following policy *π*, LSTD uses ${\hat{\Phi }}_{t}=[\phi (s_{1}),\ldots ,\phi (s_{t}){]}^{T}$, ${\hat{\Phi }}_{t}^{\prime }=[\phi (s_{1}^{\prime }),\ldots ,\phi (s_{t}^{\prime }){]}^{T}$, and ${\hat{R}}_{t}=[r_{1},\ldots ,r_{t}{]}^{T}$ to replace Φ, *P* ^*π*^Φ, and *R* ^*π*^, respectively. Then, (5) can be approximately rewritten as

(6) $$\begin{array}{c} w_{t}=\tilde{f}\left(\;w_{t}\;\right)=\underset{u\in R^{m}}{\arg\min}{\left\|\;{\hat{\Phi }}_{t}u-\left(\;{\hat{R}}_{t}+\gamma {\hat{\Phi }}_{t}^{\prime }w_{t}\;\right)\;\right\|}_{2}^{2}. \end{array}$$

Let $\partial {\left\|{\hat{\Phi }}_{t}u-\left({\hat{R}}_{t}+\gamma {\hat{\Phi }}_{t}^{\prime }w_{t}\right)\right\|}_{2}^{2}/\partial u=0$; we have

(7) $$\begin{array}{c} \tilde{f}\left(\;w_{t}\;\right)=u^{*}={\left(\;{\hat{\Phi }}_{t}^{T}{\hat{\Phi }}_{t}\;\right)}^{-1}{\hat{\Phi }}_{t}^{T}\left(\;{\hat{R}}_{t}+\gamma {\hat{\Phi }}_{t}^{\prime }w_{t}\;\right). \end{array}$$

Thus, the fixed point $w_{t}=\tilde{f}(w_{t})$ can be found by

(8) $$\begin{array}{c} w_{t}={\left(\;{\hat{\Phi }}_{t}^{T}\left(\;{\hat{\Phi }}_{t}-\gamma {\hat{\Phi }}_{t}^{\prime }\;\right)\;\right)}^{-1}{\hat{\Phi }}_{t}^{T}{\hat{R}}_{t}. \end{array}$$

## 3. Regularized OSKRLSTD Algorithms

To overcome the weaknesses of the previous kernel-based LSTD algorithms, we propose two regularized OSKRLSTD algorithms in this section.

### 3.1. OSKRLSTD-*L*_2_ Algorithm

Now, we use *L*_2_ regularization and online sparsification to derive the first OSKRLSTD algorithm, which is called OSKRLSTD-*L*_2_.

First, we use the kernel trick to kernelize (6). Suppose the feature dictionary *𝒟*_*t*_ = {**d**_*j*_∣**d**_*j*_ ∈ *𝒮*, *j* = 1,…, *n*_*t*_}, and let Φ_*t*_ = [**ϕ**(**d**_1_),…, **ϕ**(**d**_*n*_*t*__)]^*T*^ denote the corresponding feature matrix. By the Representer Theorem [24], **w**_*t*_ and **u** can be expressed as follows:

(9) wt=ΦtTαt=∑j=1ntαjϕdj,u=ΦtTβ=∑j=1ntβjϕdj,

where **α**_*t*_ = [*α*_*t*,1_,…, *α*_*t*,*n*_*t*__]^*T*^ and **β** = [*β*_1_,…, *β*_*n*_*t*__]^*T*^ are the coefficient vector of **w**_*t*_ and **u**, respectively. Then, from (6), we have

(10) $$\begin{array}{c} \alpha_{t}=\hat{f}\left(\;\alpha_{t}\;\right)=\underset{\beta \in R^{n_{t}}}{\arg\min}{\left\|\;{\hat{\Phi }}_{t}\Phi_{t}^{T}\beta -\left(\;{\hat{R}}_{t}+\gamma {\hat{\Phi }}_{t}^{\prime }\Phi_{t}^{T}\alpha_{t}\;\right)\;\right\|}_{2}^{2}. \end{array}$$

By the Mercer Theorem [24], the inner product of two feature vectors can be calculated by *k*(**s**_*i*_, **s**_*j*_) = **ϕ**(**s**_*i*_)^*T*^ **ϕ**(**s**_*j*_). Thus, we can define **K**_*t*_ = Φ_*t*_Φ_*t*_^*T*^, ${\hat{K}}_{t}=\Phi_{t}{\hat{\Phi }}_{t}^{T}$, and ${\hat{K}}_{t}^{\prime }=\Phi_{t}({\hat{\Phi }}_{t}^{\prime }{)}^{T}$. On this basis, (10) can be rewritten as

(11) $$\begin{array}{c} \alpha_{t}=\hat{f}\left(\;\alpha_{t}\;\right)=\underset{\beta \in R^{n_{t}}}{\arg\min}{\left\|\;{\hat{K}}_{t}^{T}\beta -\left(\;{\hat{R}}_{t}+\gamma {\left(\;{\hat{K}}_{t}^{\prime }\;\right)}^{T}\alpha_{t}\;\right)\;\right\|}_{2}^{2}. \end{array}$$

Second, we try to derive the *L*_2_-regularized solution of (11). Add an *L*_2_-norm penalty into (11); that is,

(12) αtf^αt=arg⁡minβ∈Rnt⁡K^tTβ−R^t+γK^t′Tαt22+ηβ22,

where *η* ∈ [0, *∞*) is a regularization parameter. Let $\partial ({\left\|{\hat{K}}_{t}^{T}\beta -\left({\hat{R}}_{t}+\gamma {\left({\hat{K}}_{t}^{\prime }\right)}^{T}\alpha_{t}\right)\right\|}_{2}^{2}+\eta {\left\|\beta \right\|}_{2}^{2})/\partial \beta =0$; we have

(13) $$\begin{array}{c} {\hat{K}}_{t}\left(\;{\hat{K}}_{t}^{T}\beta^{*}-\gamma {\left(\;{\hat{K}}_{t}^{\prime }\;\right)}^{T}\alpha_{t}\;\right)+\eta \beta^{*}={\hat{K}}_{t}{\hat{R}}_{t}. \end{array}$$

Since **w**_*t*_ = **u** ^*∗*^, we easily have **α**_*t*_ = **β** ^*∗*^ from (9). Then, the above equation can be rewritten as

(14) $$\begin{array}{c} \left(\;{\hat{K}}_{t}{\left(\;{\hat{K}}_{t}-\gamma {\hat{K}}_{t}^{\prime }\;\right)}^{T}+\eta I_{t}\;\right)\alpha_{t}={\hat{K}}_{t}{\hat{R}}_{t}, \end{array}$$

where **I**_*t*_ is the *n*_*t*_ × *n*_*t*_ identity matrix. Thus, **α**_*t*_ can be analytically solved as

(15) $$\begin{array}{c} \alpha_{t}={\left(\;{\hat{K}}_{t}{\left(\;{\hat{K}}_{t}-\gamma {\hat{K}}_{t}^{\prime }\;\right)}^{T}+\eta I_{t}\;\right)}^{-1}{\hat{K}}_{t}{\hat{R}}_{t}=A_{t}^{-1}b_{t}, \end{array}$$

where **A**_*t*_ ∈ *ℝ* ^*n*_*t*_×*n*_*t*_^ and **b**_*t*_ ∈ *ℝ* ^*n*_*t*_^ denote

(16) AtK^tK^t−γK^t′T+ηIt=∑i=1tktsiΔktTsi,si′+ηIt,btK^tR^t=∑i=1tktsiri,

where **k**_*t*_(·) = [*k*(·, **d**_1_),…, *k*(·, **d**_*n*_*t*__)]^*T*^ and Δ**k**_*t*_(**s**_*i*_, **s**_*i*_′) = **k**_*t*_(**s**_*i*_) − *γ* **k**_*t*_(**s**_*i*_′).

Third, we derive the recursive formulas of **A**_*t*_^−1^ and **α**_*t*_. Under online sparsification, there are two cases: (1) *𝒟*_*t*_ = *𝒟*_*t*−1_, *n*_*t*_ = *n*_*t*−1_, **k**_*t*_(·) = **k**_*t*−1_(·), Δ**k**_*t*_(**s**_*i*_, **s**_*i*_′) = Δ**k**_*t*−1_(**s**_*i*_, **s**_*i*_′), and **I**_*t*_ = **I**_*t*−1_; (2) *𝒟*_*t*_ = *𝒟*_*t*−1_ ∪ {**s**_*t*_}, *n*_*t*_ = *n*_*t*−1_ + 1, **k**_*t*_(·) = [**k**_*t*−1_^*T*^(·), *k*(·, **s**_*t*_)]^*T*^, Δ**k**_*t*_(**s**_*i*_, **s**_*i*_′) = [Δ**k**_*t*−1_^*T*^(**s**_*i*_, **s**_*i*_′), Δ*k*_*t*_(**s**_*i*_, **s**_*i*_′)]^*T*^, where Δ*k*_*t*_(**s**_*i*_, **s**_*i*_′) = *k*(**s**_*i*_, **s**_*t*_) − *γk*(**s**_*i*_′, **s**_*t*_), and **I**_*t*_ is expanded as

(17) $$\begin{array}{c} I_{t}=\left[\begin{array}{cc} I_{t-1} & 0_{t-1}\\ 0_{t-1}^{T} & 1 \end{array}\right], \end{array}$$

where 0_*t*−1_ is the *n*_*t*−1_ dimensional zero vector.

For the first case, (16) can be rewritten as follows:

(18) At=At−1+kt−1stΔkt−1Tst,st′,

(19) bt=bt−1+kt−1strt.

Applying the matrix-inversion lemma [25] for **A**_*t*_^−1^, we get

(20) $$\begin{array}{c} A_{t}^{-1}=A_{t-1}^{-1}-\frac{A_{t-1}^{-1}k_{t-1}\left(s_{t}\right)\Delta k_{t-1}^{T}\left(s_{t},s_{t}^{\prime }\right)A_{t-1}^{-1}}{1+\Delta k_{t-1}^{T}\left(s_{t},s_{t}^{\prime }\right)A_{t-1}^{-1}k_{t-1}\left(s_{t}\right)}. \end{array}$$

Thus, plugging (19) and (20) into (15), we obtain

(21) $$\begin{array}{c} \alpha_{t}=\alpha_{t-1}+\frac{\left(r_{t}-\alpha_{t-1}^{T}\Delta k_{t-1}\left(s_{t},s_{t}^{\prime }\right)\right)A_{t-1}^{-1}k_{t-1}\left(s_{t}\right)}{1+\Delta k_{t-1}^{T}\left(s_{t},s_{t}^{\prime }\right)A_{t-1}^{-1}k_{t-1}\left(s_{t}\right)}. \end{array}$$

For the second case, (16) can be rewritten as follows:

(22) At=A~thtgtTpt,bt=b~tqt,

where ${\tilde{A}}_{t}$ and ${\tilde{b}}_{t}$ are the same as the updated **A**_*t*_ and **b**_*t*_ when the feature dictionary keeps unchanged, **h**_*t*_ = ∑_*i*=1_^*t*^Δ*k*_*t*_(**s**_*i*_, **s**_*i*_′)**k**_*t*−1_(**s**_*i*_), **g**_*t*_ = ∑_*i*=1_^*t*^ *k*(**s**_*i*_, **s**_*t*_)Δ**k**_*t*−1_(**s**_*i*_, **s**_*i*_′), *p*_*t*_ = ∑_*i*=1_^*t*^ *k*(**s**_*i*_, **s**_t_)Δ*k*_*t*_(**s**_*i*_, **s**_*i*_′) + *η*, and *q*_*t*_ = ∑_*i*=1_^*t*^ *k*(**s**_*i*_, **s**_*t*_)*r*_*i*_. However, computing **h**_*t*_, **g**_*t*_, *p*_*t*_, and *q*_*t*_ requires caching all history samples, and the computational cost will become more and more expensive as *t* increases. Inspired by the work of Van Vaerenbergh et al. [26], we introduce a sliding window *ℋ*_*t*_ to deal with these problems. Let *ℋ*_*t*_ = {(**s**_*j*_, **s**_*j*_′, *r*_*j*_)∣*j* = max⁡(1, *t* − *M* + 1),…, *t*}, where *M* is the window size. We only use the samples in *ℋ*_*t*_ to evaluate **h**_*t*_, **g**_*t*_, *p*_*t*_, and *q*_*t*_; that is,

(23) h~t=∑j∈HtΔktsj,sj′kt−1sj,g~t=∑j∈Htksj,stΔkt−1sj,sj′,p~t=∑j∈Htksj,stΔktsj,sj′+η,q~t=∑j∈Htksj,strj.

Then, similar to those in the first case, **A**_*t*_^−1^ and **α**_*t*_ can be derived as follows:

(24) At−1=1mtmtA~t−1+A~t−1h~tg~tTA~t−1−A~t−1h~t−g~tTA~t−11,

(25) αt=1mtmtα~t−A~t−1h~tq~t−g~tTα~tq~t−g~tTα~t,

where $m_{t}={\tilde{p}}_{t}-{\tilde{g}}_{t}^{T}{\tilde{A}}_{t}^{-1}{\tilde{h}}_{t}$ and ${\tilde{\alpha }}_{t}$ is the same as the updated **α**_*t*_ when the dictionary keeps unchanged.

Finally, we summarize the whole algorithm in Algorithm 1.

**Remark 1 .**

Here, we do not restrict the OSKRLSTD-*L*_2_ algorithm to a specific online sparsification method. That means it can be combined with many popular sparsification methods such as the novelty criterion (NC) [27] and the ALD criterion.

**Remark 2 .**

Although the OSKRLSTD-*L*_2_ algorithm is designed for infinite horizon tasks, it can be modified for episodic tasks. When **s**_*t*_′ is an absorbing state, it only requires setting *γ* = 0 temporarily and setting **s**_*t*+1_ as the start state of next episode.

**Remark 3 .**

Our simulation results show that a big sliding window cannot help improve the convergence performance of the OSKRLSTD-*L*_2_ algorithm. Thus, to save memory and reduce the computational cost, *M* should be set to a small integer.

### 3.2. OSKRLSTD-*L*_1_ Algorithm

In this subsection, we use *L*_1_ regularization and online sparsification to derive the second OSKRLSTD algorithm, which is called OSKRLSTD-*L*_1_.

First, we try to derive the *L*_1_-regularized solution of (11). Add an *L*_1_-norm penalty into (11); that is,

(26) αtf^αt=arg⁡minβ∈Rnt⁡K^tTβ−R^t+γK^t′Tαt22+2ξβ1,

where *ξ* ∈ [0, *∞*) is a regularization parameter. However, ‖**β**‖_1_ is not differentiable. Similar to Painter-Wakefield and Parr in [28], we resort to the subdifferential of $g(\beta )={\left\|{\hat{K}}_{t}^{T}\beta -({\hat{R}}_{t}+\gamma ({\hat{K}}_{t}^{\prime }{)}^{T}\alpha_{t})\right\|}_{2}^{2}+2\xi {\left\|\beta \right\|}_{1}$; that is,

(27) ∇gβ=2K^tK^tTβ−R^t+γK^t′Tαt+2ξsgn⁡β,

where sgn⁡(**β**) is the set-valued function defined component-wise as

(28) $$\begin{array}{c} \mathrm{sgn}\left(\;\beta_{j}\;\right)=\left\{\begin{array}{cc} \left\{+1\right\} & \beta_{j}>0\\ \left[-1,+1\right] & \beta_{j}=0\\ \left\{-1\right\} & \beta_{j}<0. \end{array}\right. \end{array}$$

Let ∇*g*(**β**) = 0, so that

(29) $$\begin{array}{c} {\hat{K}}_{t}\left(\;{\hat{K}}_{t}^{T}\beta^{*}-\gamma {\left(\;{\hat{K}}_{t}^{\prime }\;\right)}^{T}\alpha_{t}\;\right)={\hat{K}}_{t}{\hat{R}}_{t}-\xi \mathrm{sgn}\left(\;\beta^{*}\;\right). \end{array}$$

Since **w**_*t*_ = **u** ^*∗*^, we also have **α**_*t*_ = **β** ^*∗*^ from (9). Then, the above equation can be rewritten as

(30) $$\begin{array}{c} {\hat{K}}_{t}{\left(\;{\hat{K}}_{t}-\gamma {\hat{K}}_{t}^{\prime }\;\right)}^{T}\alpha_{t}={\hat{K}}_{t}{\hat{R}}_{t}-\xi \mathrm{sgn}\left(\;\alpha_{t}\;\right), \end{array}$$

where sgn⁡(**α**_*t*_) has the same meaning as sgn⁡(**β**). To avoid the singularity of ${\hat{K}}_{t}({\hat{K}}_{t}-\gamma {\hat{K}}_{t}^{\prime }{)}^{T}$ and further reduce the complexity of the subsequent derivation, we introduce *η* **α**_*t*_ into both sides; that is,

(31) K^tK^t−γK^t′T+ηItαt=K^tR^t+ηαt−ξsgn⁡αt,

where *η* ∈ [0, *∞*) is a regularization parameter. Obviously, the left hand side of (31) is the same as that of (14). Thus, from (16), the above equation can be rewritten as

(32) $$\begin{array}{c} A_{t}\alpha_{t}=b_{t}+\eta \alpha_{t}-\xi \mathrm{sgn}\left(\;\alpha_{t}\;\right). \end{array}$$

Then, we have the following fixed-point equation:

(33) $$\begin{array}{c} \alpha_{t}=\mu_{t}+A_{t}^{-1}\left(\;\eta \alpha_{t}-\xi \mathrm{sgn}\left(\;\alpha_{t}\;\right)\;\right), \end{array}$$

where ***μ***_*t*_ denotes

(34) $$\begin{array}{c} \mu_{t}=A_{t}^{-1}b_{t}. \end{array}$$

Unfortunately, here, **α**_*t*_ cannot be solved analytically.

Second, we investigate how to find the fixed point of (33). In *L*_1_-regularized LSTD algorithms [5, 29], researchers often used the LASSO method to tackle this problem. However, the LASSO method is inherently a batch method and is unsuitable for online learning. Instead, we resort to the fixed-point subiteration method introduced in [13]. We first use the sign function sign⁡(**α**_*t*_) to replace sgn⁡(**α**_*t*_) in (33). Then, we can construct the following subiteration:

(35) $$\begin{array}{c} \alpha_{t}^{l+1}=\mu_{t}+A_{t}^{-1}\left(\;\eta \alpha_{t}^{l}-\xi \mathrm{sign}\left(\;\alpha_{t}^{l}\;\right)\;\right), \end{array}$$

where *l* ∈ *ℕ* ^+^ denotes the *l*th subiteration and **α**_*t*_^1^ is initialized to ***μ***_*t*_ since the fixed point will be close to ***μ***_*t*_ if *η* and *ξ* are small. If the subiteration number reaches a preset value *N* ∈ *ℕ* ^+^ or ‖**α**_*t*_^*v*+1^ − **α**_*t*_^*v*^‖ is less than or equal to a preset threshold *ε* ∈ *ℝ* ^+^, the subiteration will stop. From (32) and (28), if |(**b**_*t*_ + *η* **α**_*t*_ − **A**_*t*_ **α**_*t*_)_*j*_ | < *ξ*, *α*_*t*,*j*_ should be 0. Obviously, the replacement of sgn⁡(**α**_*t*_) makes **α**_*t*_ lose the ability to select features. To remedy this situation, after the whole subiteration, we remove the weakly dependent elements from *𝒟*_*t*_ according to the magnitude of **α**_*t*_; that is,

(36) $$\begin{array}{c} D_{t}=\Psi_{I_{t}}\left(\;D_{t}\;\right), \end{array}$$

where Ψ_*ℐ*_*t*__(·) denotes the operation to remove the elements indexed by the set *ℐ*_*t*_, which is determined by

(37) $$\begin{array}{c} I_{t}=\left\{\;j\;|\;-v\leq \alpha_{t,j}\leq v,\;\;j=1,\ldots ,n_{t}-1\;\right\}, \end{array}$$

where *v* ∈ *ℝ* ^+^ is a preset threshold. Note that we do not remove the last element **d**_*n*_*t*__ of *𝒟*_*t*_, since |*α*_*n*_*t*__| is probably very small, especially when **d**_*n*_*t*__ is just added to *𝒟*_*t*_. Similarly, we perform Ψ_*ℐ*_*t*__(**α**_*t*_) and Ψ_*ℐ*_*t*__(***μ***_*t*_) to remove the weakly dependent coefficients. From (16), **A**_*t*_^−1^ also requires removing some rows and columns. Unfortunately, we cannot use the method in [30] to do this like Chen et al. in [13], since **A**_*t*_^−1^ is not a symmetry matrix. Considering that **b**_*t*_ will remove the corresponding elements if *𝒟*_*t*_ is pruned, we directly perform Ψ_*ℐ*_*t*__(**A**_*t*_^−1^) to remove the rows and columns indexed by *ℐ*_*t*_. Although this method may bring some bias into **A**_*t*_^−1^, our simulation results show that it is feasible and effective. The whole fixed-point subiteration and online pruning algorithm are summarized in Algorithm 2.

**Remark 4 .**

Our simulation results show that Algorithm 2 will converge in few iterations. Thus, Algorithm 2 does not become the computational bottleneck of the OSKRLSTD-*L*_1_ algorithm, and the maximum subiteration number *N* can be set to a small positive integer.

Third, we derive the recursive formulas of **A**_*t*_^−1^ and ***μ***_*t*_. Although the dictionary can be pruned by using Algorithm 2, it still has the risk of rapidly growing if new samples are allowed to be added continually. Thus, the conventional sparsification method is also required to be considered here. Similar to Section 3.1, there are two cases under online sparsification. Since **A**_*t*_ and ***μ***_*t*_ have the same definitions as **A**_*t*_ and **α**_*t*_ in the OSKRLSTD-*L*_2_ algorithm, we can directly use (20) and (24) for updating **A**_*t*_^−1^ and rewrite (21) and (25) for updating ***μ***_*t*_. If **s**_*t*_ dissatisfies the sparsification condition, ***μ***_*t*_ will be updated by

(38) $$\begin{array}{c} \mu_{t}=\mu_{t-1}+\frac{\left(r_{t}-\mu_{t-1}^{T}\Delta k_{t-1}\left(s_{t},s_{t}^{\prime }\right)\right)A_{t-1}^{-1}k_{t-1}\left(s_{t}\right)}{1+\Delta k_{t-1}^{T}\left(s_{t},s_{t}^{\prime }\right)A_{t-1}^{-1}k_{t-1}\left(s_{t}\right)}. \end{array}$$

Otherwise, ***μ***_*t*_ will be updated by

(39) $$\begin{array}{c} \mu_{t}=\frac{1}{m_{t}}\left[\begin{array}{c} m_{t}{\tilde{\mu }}_{t}-{\tilde{A}}_{t}^{-1}{\tilde{h}}_{t}\left({\tilde{q}}_{t}-{\tilde{g}}_{t}^{T}{\tilde{\mu }}_{t}\right)\\ {\tilde{q}}_{t}-{\tilde{g}}_{t}^{T}{\tilde{\mu }}_{t} \end{array}\right], \end{array}$$

where ${\tilde{h}}_{t}$, ${\tilde{g}}_{t}$, ${\tilde{p}}_{t}$, and ${\tilde{q}}_{t}$ are also calculated by (23). Since *𝒟*_*t*_, **A**_*t*_^−1^, and ***μ***_*t*_ will be pruned by Algorithm 2 after the update, it is important to note that ${\tilde{A}}_{t}^{-1}$ and ${\tilde{\mu }}_{t}$ in (39) denote **A**_*t*_^−1^ and ***μ***_*t*_ updated by *𝒟*_*t*−1_ but unpruned by Ψ_*ℐ*_*t*__(·). Likewise, when (24) is used here, ${\tilde{A}}_{t}^{-1}$ has the same meaning.

Finally, we summarize the whole algorithm in Algorithm 3. For episodic tasks, the modification is the same as Remark 2. In addition, similar to Remark 3, the sliding-window size *M* should also be set to a small integer.

**Remark 5 .**

By pruning the weakly dependent features, the OSKRLSTD-*L*_1_ algorithm can yield a much sparser solution than the OSKRLSTD-*L*_2_ algorithm.

## 4. Simulations

In this section, we use a nonnoise chain and a noise chain [2, 20, 31] to demonstrate the effectiveness of our proposed algorithms. For comparison purposes, RLSTD [1] and SKRLSTD [21] algorithms are also tested in the simulations. To analyze the effect of regularization and online pruning on the performance of our algorithms, the OSKRLSTD-*L*_2_ algorithm with *η* = 0 and the OSKRLSTD-*L*_1_ algorithm with *v* = 0 (called OSKRLSTD-0 and OSKRLSTD-*L*_1*u*_, resp.) are tested here, too. In addition, the effects of the sliding-window size on the performance of our algorithms and OSKRLSTD-*L*_1*u*_ are evaluated as well.

### 4.1. Simulation Settings

**Figure 1:**
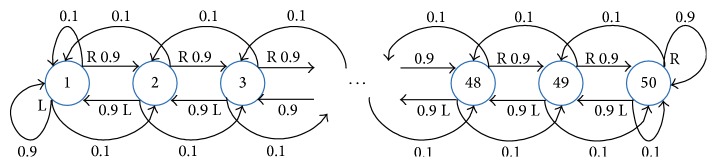
The 50-state chain problem.

As shown in Figure 1, in both chain problems, each chain consists of 50 states, which are numbered from 1 to 50. For each state, there are two actions available, that is, “left” (L) and “right” (R). Each action succeeds with probability 0.9, changing the state in the intended direction, and fails with probability 0.1, changing the state in the opposite direction. The two boundaries of each chain are dead-ends, and the discount factor *γ* of each chain is set to 0.9. For the nonnoise chain, the reward is 1 only in states 10 and 41, whereas, for the noise chain, the reward is corrupted by an additive Gaussian noise 0.3*𝒩*(0,1). Due to the symmetry, the optimal policy for both chains is to go right in states 1–9 and 26–41 and left in states 10–25 and 42–50. Here, we use it as the policy *π* to be evaluated. Note that the state transition probabilities are available only for solving the true state-value functions *V* ^*π*^, and they are assumed to be unknown for all algorithms compared here.

In the implementations of all tested algorithms for both chain problems, the settings are summarized as follows: (i) For all OSKRLSTD algorithms, the Mercer kernel is defined as *k*(**x**, **y**) = exp⁡(−‖**x** − **y**‖^2^/16), the sparsification condition is defined as min_**d**_*j*_∈*𝒟*_*t*−1__‖**s**_*t*_ − **d**_*j*_‖ > 2, and the sliding-window size *M* is set to 5. Besides, for the OSKRLSTD-*L*_1_ algorithm, the regularization parameters *η* and *ξ* are set to 0.8 and 0.3, the maximum subiteration number *N* is set to 10, the precision threshold *ε* is set to 0.1, and the pruning threshold *v* is set to 0.4; for the OSKRLSTD-*L*_1*u*_ algorithm, *η*, *ξ*, and *N* are the same as those in the OSKRLSTD-*L*_1_ algorithm; for the OSKRLSTD-*L*_2_ algorithm, *ξ* is set to 1. (ii) For the SKRLSTD algorithm, the Mercer kernel and the sparsification condition are the same as those in each OSKRLSTD algorithm. (iii) For the RLSTD algorithm, the feature vector **ϕ**(**s**) consists of 19 Gaussian radius basis functions (GRBFs) plus a constant term 1, resulting in a total of 20 basis functions. The GRBF has the same definition as the Mercer kernel used in each OSKRLSTD algorithm, and the centers of GRBFs are uniformly distributed over [1,50]. In addition, the variance matrix *C*_0_ of RLSTD is initialized to 0.4**I**, where **I** is the 20 × 20 identity matrix. (iv) In the simulations, each algorithm performs 50 runs, each run includes 100 episodes, and each episode is truncated after 100 time steps. In particular, the SKRLSTD algorithm requires an extra run for offline sparsification before each regular run.

### 4.2. Simulation Results

**Figure 2:**
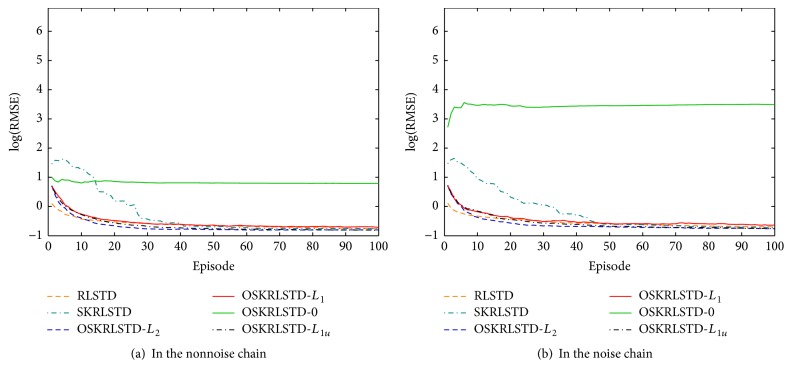
Learning curves of all tested algorithms.

**Figure 3:**
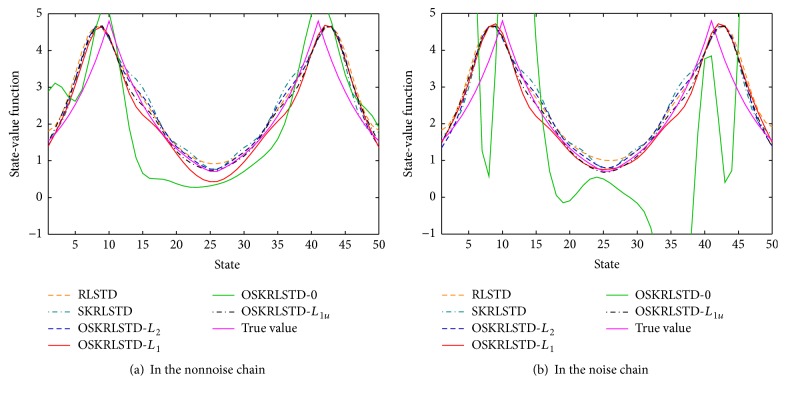
${\hat{V}}^{\pi }(s)$ approximated by all tested algorithms at the final episode.

**Figure 4:**
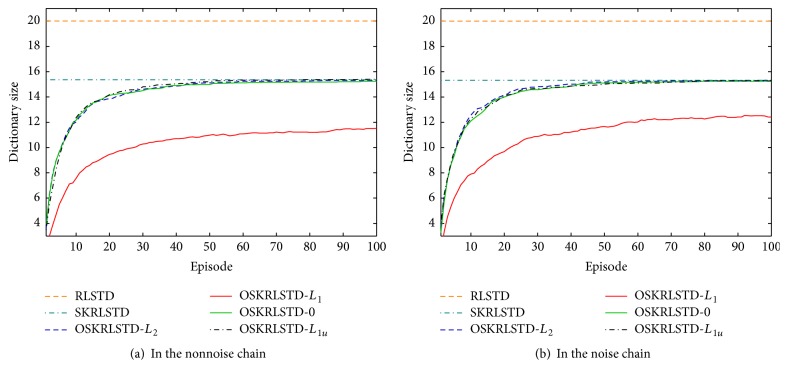
Dictionary growth curves of all tested algorithms.

**Figure 5:**
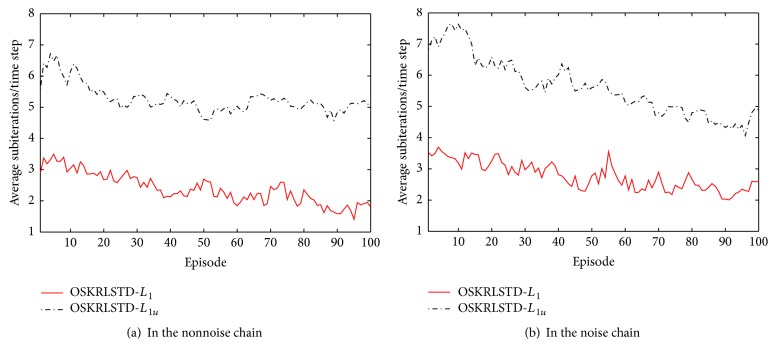
Average subiterations in OSKRLSTD-*L*_1_ and OSKRLSTD-*L*_1*u*_ algorithms.

We first report the comparison results of all tested algorithms with the simulation settings described in Section 4.1. Their learning curves are shown in Figure 2. At each episode, the root mean square error (RMSE) of each algorithm is calculated by $RMSE=(1/50)\sum_{j=1}^{50}((1/50)\sum_{s=1}^{50}({\hat{V}}_{j}^{\pi }(s)-V^{\pi }(s){)}^{2}{)}^{0.5}$, where *V* ^*π*^(**s**) is solved by (1) and ${\hat{V}}_{j}^{\pi }(s)$ is the approximate value of the *j*th run. From Figure 2, we can observe that (i) OSKRLSTD-*L*_2_ and OSKRLSTD-*L*_1_ can obtain the similar performance as RLSTD and converge much faster than SKRLSTD. (ii) Without regularization, the performance of OSKRLSTD-0 becomes very poor, especially in the noise chain. In contrast, OSKRLSTD-*L*_2_ and OSKRLSTD-*L*_1_ still perform well. (iii) The performance of OSKRLSTD-*L*_1*u*_ is only slightly better than that of OSKRLSTD-*L*_1_, which indicates that online pruning has little effect on the performance.  Figure 3 illustrates ${\hat{V}}^{\pi }(s)$ approximated by all tested algorithms at the final episode. Clearly, OSKRLSTD-0 has lost the ability to approximate *V* ^*π*^(**s**) of the noise chain.  Figure 4 shows the dictionary growth curves of all tested algorithms. Compared with RLSTD and SKRLSTD, all OSKRLSTD algorithms can construct the dictionary automatically, and OSKRLSTD-*L*_1_ yields a much sparser dictionary.  Figure 5 shows the average subiterations per time step in OSKRLSTD-*L*_1_ and OSKRLSTD-*L*_1*u*_. As episodes increase, the subiterations decline gradually. In addition, online pruning can reduce the subiterations significantly. Even in the noise chain, the subiterations are small. Finally, the main simulation results of all tested algorithms at the final episode are summarized in Table 1.

**Figure 6:**
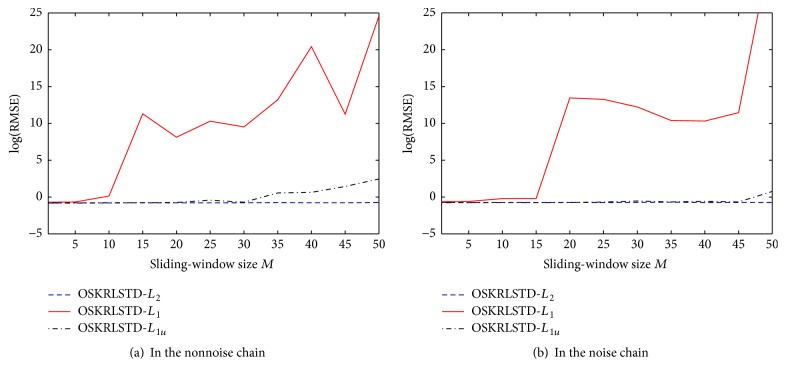
Effect of the sliding-window size *M* on three OSKRLSTD algorithms.

**Figure 7:**
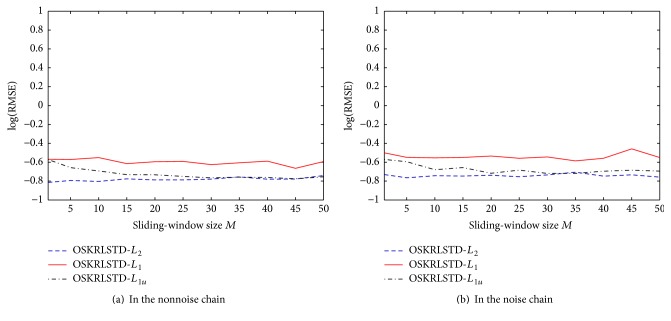
Effect of the sliding-window size *M* on three OSKRLSTD algorithms with new parameters.

Next, we evaluate the effect of the sliding-window size on our proposed algorithms and OSKRLSTD-*L*_1*u*_ with *M* = 1,5, 10,…, 45,50. The logarithmic RMSEs of each algorithm at the final episode are illustrated in Figure 6. Note that the parameter settings of these algorithms are the same as those described in Section 4.1 except for *M*. From Figure 6, OSKRLSTD-*L*_1_ and OSKRLSTD-*L*_1*u*_ obviously become worse rather than better as the window size increases, and OSKRLSTD-*L*_2_ has a strong adaptability to different window sizes. The reason for this result is analyzed as follows: From the derivation of our algorithms, the influence of the window size is mainly manifest in **A**_*t*_^−1^. Since here **A**_*t*_^−1^ is calculated by recursive update instead of matrix inversion and samples are used one by one, using too many history samples together may increase the calculation error. In OSKRLSTD-*L*_2_, a moderate regularization parameter *η* can relieve the influence of this error. In contrast, in OSKRLSTD-*L*_1_ and OSKRLSTD-*L*_1*u*_, the subiteration may expand the influence. Especially for OSKRLSTD-*L*_1_, online pruning can introduce the new error, which further worsens the convergence performance. To verify the above analysis, we reset *η* = 0.6, *ξ* = 0.3, and *N* = 1 for OSKRLSTD-*L*_1_ and OSKRLSTD-*L*_1*u*_ and reevaluate the effect of the window size. The new results are illustrated in Figure 7. As expected, OSKRLSTD-*L*_1_ and OSKRLSTD-*L*_1*u*_ can also adapt to *M*. Nevertheless, there is still no proof that a big window size can help improve the convergence performance of OSKRLSTD-*L*_2_ and OSKRLSTD-*L*_1_. Thus, as stated in Remark 3, *M* is suggested to be set to a small integer in practice.

## 5. Conclusion

As an important approach for policy evaluation, LSTD algorithms can use samples more efficiently and eliminate all step-size parameters. But they require users to design the feature vector manually and often require many features to approximate state-value functions. Recently, there are some works on these issues by combining with sparse kernel methods. However, these works do not consider regularization and their sparsification processes are batch or offline. In this paper, we propose two online sparse kernel recursive least-squares TD algorithms with *L*_2_ and *L*_1_ regularization, that is, OSKRLSTD-*L*_2_ and OSKRLSTD-*L*_1_. By using Bellman operator along with projection operator, our derivation is more simple. By combining online sparsification, *L*_2_ and *L*_1_ regularization, recursive least squares, a sliding window, and the fixed-point subiteration, our algorithms not only can construct the feature dictionary online but also can avoid overfitting and eliminate the influence of noise. These advantages make them more suitable for online RL problems with a large or continuous state space. In particular, compared with the OSKRLSTD-*L*_2_ algorithm, the OSKRLSTD-*L*_1_ algorithm can yield a much sparser dictionary. Finally, we illustrate the performance of our algorithms and compare them with RLSTD and SKRLSTD algorithms by several simulations.

There are also some interesting topics to be studied in future work: (i) How to select proper regularization parameter should be investigated. (ii) A more thorough simulation analysis is needed, including an extension of our algorithms to learning control problems. (iii) Eligibility traces would be combined for further improving the performance of our algorithms. (iv) The convergence and prediction error bounds of our algorithms will be analyzed theoretically.

## Figures and Tables

**Algorithm 1:**
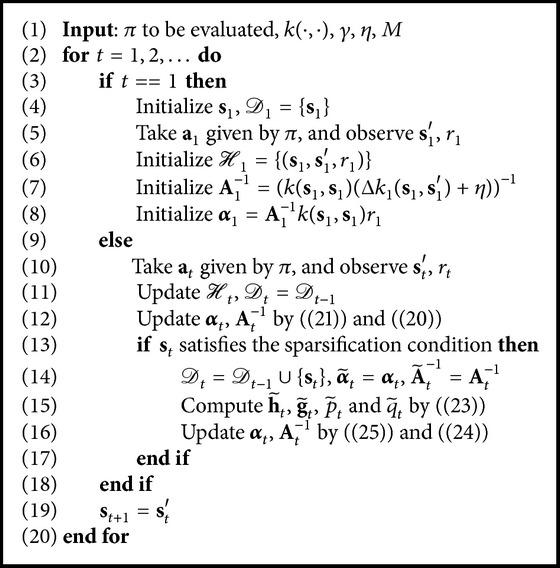
OSKRLSTD-*L*_2_.

**Algorithm 2:**
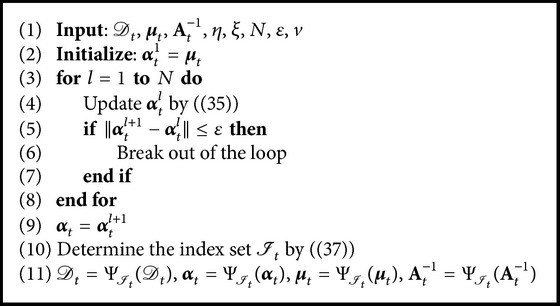
Fixed-point subiteration and online pruning.

**Algorithm 3:**
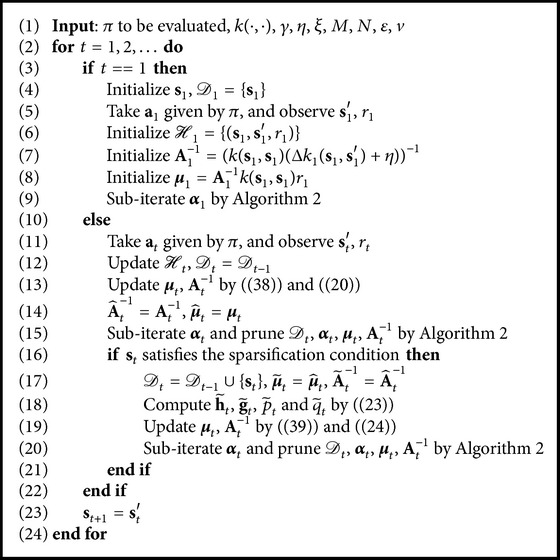
OSKRLSTD-*L*_1_.

**Table 1:** Main simulation results on both chains at the final episode.

Algorithm	Nonnoise chain			Noise chain		
	RMSE	Dictionary size	Subiterations	RMSE	Dictionary size	Subiterations
RLSTD	0.47 ± 0.03	20	—	0.50 ± 0.04	20	—
SKRLSTD	0.47 ± 0.05	15.36 ± 0.78	—	0.49 ± 0.06	15.32 ± 0.71	—
OKRLSTD-*L*_2_	0.45 ± 0.05	15.30 ± 0.81	—	0.47 ± 0.04	15.32 ± 0.84	—
OKRLSTD-*L*_1_	0.49 ± 0.08	11.52 ± 1.16	1.81 ± 1.82	0.53 ± 0.10	12.42 ± 1.13	2.60 ± 2.56
OKRLSTD-0	2.21 ± 0.05	15.25 ± 0.87	—	32.92 ± 68.67	15.24 ± 0.77	—
OKRLSTD-*L*_1*u*_	0.44 ± 0.05	15.40 ± 0.76	5.08 ± 3.24	0.47 ± 0.05	15.28 ± 0.88	4.90 ± 3.26
